# Bronchoscopic intervention in the management of primary tracheobronchial adenoid cystic carcinoma with dyspnea

**DOI:** 10.3389/fonc.2025.1643239

**Published:** 2025-09-12

**Authors:** Xiuxiu Wu, Xiaojian Qiu, Beibei Jin, Jinmu Niu, Yankun Jin, Xiaoning Bu, Juan Wang

**Affiliations:** Department of Respiratory and Critical Care Medicine, Beijing Tiantan Hospital, Capital Medical University, Beijing, China

**Keywords:** tracheobronchial adenoid cystic carcinoma, bronchoscopic intervention, airway stenosis, progression-free survival, overall survival

## Abstract

**Background:**

Primary tracheobronchial adenoid cystic carcinoma (TACC) is a rare, slow-progressing malignant tumor whose airway obstruction frequently compromise survival. This study aimed to investigate the value of interventional bronchoscopy in TACC.

**Methods:**

We retrospectively analyzed the clinical data of TACC patients treated between January 2006 and December 2024. 52 patients were stratified into two groups: the bronchoscopic intervention alone group (*n* = 25) and combined therapy group (*n* = 27). Kaplan-Meier methodology was employed to estimate median progression-free survival (PFS), overall survival (OS), and survival rates. Prognostic factors were further evaluated using Cox proportional hazards model.

**Results:**

The mean age of 52 TACC patients was 51.77 ± 14.40 years. Bronchoscopic intervention demonstrated significant reduction in mMRC score and Freitag grade (*P* < 0.01). Median PFS and OS were 20 months and 118 months, respectively. The 3-/5-/10-year OS were 80.00%, 69.12%, and 34.87%. The combined therapy group exhibited superior PFS compared to the bronchoscopic intervention alone group (*P* < 0.05). Cox analysis identified combined therapy and stenosis degree as independent prognostic factors for PFS (*P* < 0.05).

**Conclusions:**

Bronchoscopic intervention demonstrated significant efficacy in alleviating dyspnea and airway stenosis while sustaining long-term survival in TACC patients. Multimodal therapy showed potential advantages in delaying disease progression.

## 1 Introduction

Primary tracheobronchial adenoid cystic carcinoma (TACC), a rare low-grade malignant salivary gland-type neoplasm originating from tracheobronchial submucosal glands, accounts for 0.1~0.2% of pulmonary malignancies (1). Characterized by an indolent growth pattern yet aggressive biological behavior, TACC exhibits longitudinal submucosal infiltration with frequent perineural and vascular invasion, predisposing patients to local recurrence and distant metastasis (2). Clinically, TACC predominantly manifests with progressive dyspnea, refractory cough, and hemoptysis due to its predilection for central airways (trachea and main bronchi). Advanced cases may develop life-threatening respiratory failure secondary to critical airway compromise. Conventional management prioritizes surgical resection (3), often combined with adjuvant chemoradiotherapy (4). However, anatomical constraints frequently preclude complete tumor extirpation, resulting in high recurrence rates and suboptimal outcomes with systemic therapies. Over recent decades, interventional bronchoscopy, including stent implantation, balloon dilation, laser, electrocautery, argon plasma coagulation (APC), and cryotherapy, have been increasingly integrated into airway stenosis management (5–7). These modalities provide immediate symptomatic relief by restoring airway patency, with emerging applications in TACC reported sporadically (8–10). Bronchoscopic interventions were primarily applied in: unresectable disease (e.g., tracheal carina involvement, extensive longitudinal tracheal involvement) or postoperative recurrence, symptom palliation for critical airway stenosis, bridge-to-surgery optimization (e.g., debulking obstructive tumors). Nevertheless, evidence regarding their short-term efficacy, durability of response, and long-term prognostic implications in TACC is scarce. This study systematically evaluates the therapeutic value of bronchoscopic intervention in TACC through multidimensional assessment of procedural outcomes, survival analyses, and prognostic factor identification. Our findings aim to establish evidence-based clinical pathways for optimizing symptom control and survival outcomes in this challenging malignancy.

## 2 Methods

### 2.1 Study population and data collection

Patients with histologically confirmed TACC treated at the Respiratory Intervention Center of Beijing Tiantan Hospital, Capital Medical University between January 2006 and December 2024 were retrospectively recruited. Inclusion criteria comprised: (1) age ≥ 18 years; (2) histopathologically confirmed TACC diagnosis; (3) completion of ≥2 bronchoscopic intervention procedures; (4) all patients signed informed consent for interventional bronchoscopy procedure. Comprehensive clinical variables were extracted from medical records, including: (1) demographics: sex, age at diagnosis, body mass index (BMI); (2) clinical profiles: medical history, smoking status, initial presenting symptoms, and imaging characteristics. (3) treatment parameters: surgical resection status, radiotherapy/chemotherapy regimens, and interventional bronchoscopy details, including anesthesia methods, ventilation strategy, classification of stenosis (location, length, type and degree), interventional modalities, and procedure related complications: hemorrhage, hypoxemia, glottic edema, hypercapnia. The primary surgical approach was sleeve resection with end-to-end anastomosis. Radiotherapy included both adjuvant postoperative radiotherapy and definitive radiotherapy, with doses ≥60 Gy. Chemotherapy regimens primarily consisted of platinum-based protocols combined with agents such as paclitaxel, and cyclophosphamide, administered at standard dosages according to NCCN guidelines.

### 2.2 Multidisciplinary team for bronchoscopic intervention

The internists conducted comprehensive preoperative evaluations to confirm indications and exclude contraindications for interventional therapy. Anesthesiologists formulated the anesthesia strategy (local anesthesia, sedation, or general anesthesia), selected appropriate anesthetic agents, and determined the ventilation approach (nasal cannula, mask, laryngeal mask, endotracheal intubation, or jet ventilation via rigid bronchoscopy) based on the patient’s clinical status. Endoscopy nurses established intravenous access and prepared surgical instruments and materials. Interventional bronchoscopists employed tailored flexible or rigid bronchoscopic techniques according to the patient’s pathology: For intraluminal stenosis, resection modalities such as laser, electrocautery (snare or probe) or cryotherapy were selected based on tumor size, morphology and vascularity. For extrinsic stenosis or mixed type, airway stents were customized according to the length of stenosis and the native tracheal diameter, followed by stent implantation at the narrowed site to alleviate obstruction.

Mild intraoperative bleeding was managed with endoscopic suction, while moderate bleeding was controlled by topical epinephrine application, iced saline irrigation, or APC. Severe hemorrhage required emergency intervention to prevent fatal asphyxiation. Postoperatively, the patient’s disposition to the intensive care unit (ICU) or general ward was determined by intraoperative complications (e.g., active airway bleeding, glottic edema), recovery of spontaneous respiration, and oxygenation stability.

### 2.3 Evaluation of short-term efficacy and safety of bronchoscopic intervention

The severity of dyspnea was assessed using the modified Medical Research Council (mMRC) Dyspnea Scale (11). The length of stenosis was measured via endoscopic evaluation or three dimensional reconstruction of cervicothoracic computed tomography (CT) imaging. Types of stenosis were categorized as intraluminal, extrinsic, or mixed type (12, 13). The degree of stenosis was quantified as the ratio of the stenotic cross-sectional area to the normal trachea or bronchus based on CT reconstruction, with specific classifications following the Freitag grading system (14): grade I (≤25%), grade II (26%–50%), grade III (51%–75%), grade IV (76%–90%), and grade V (90%-complete obstruction). In order to evaluate the short-term efficacy and safety of the procedure, changes in mMRC scores and degree of stenosis before and after the initial interventional bronchoscopy for airway stenosis were analyzed. Intraoperative complications were documented.

### 2.4 Application of bronchoscopic intervention in long-term management of TACC

All TACC patients undergoing interventional bronchoscopy procedure at our center were required to attend follow-up visits. Clinical data during follow-up, including the frequency of bronchoscopic interventions, therapeutic modalities, and procedure-related complications, were collected. Progression-free survival (PFS) and overall survival (OS) were documented. Disease status in TACC patients was assessed comprehensively based on clinical symptoms, imaging findings, and endoscopic features. In this study, disease progression was defined as meeting at least one of the following criteria: ① New or worsened dyspnea, stridor, or hemoptysis related to the primary disease (13); ② A ≥ 20% increase in the long diameter of the primary tumor on CT, according to RECIST 1.1 criteria (15); ③Endoscopic evidence of tumor enlargement, expanded obstruction, increased Freitag grade, or bronchoscopic findings of mucosal thickening and rigidity which suggesting deep invasion (16). PFS was defined as the number of months from the first bronchoscopic intervention to disease progression, while OS was defined as the number of months from disease onset to death.

### 2.5 Statistical analysis

Statistical analyses were performed using SPSS 19.0 software and GraphPad Prism 8 software. Normally distributed continuous data were expressed as mean ± standard deviation, while non-normally distributed continuous data were presented as median (interquartile range). For pre- and post-interventional bronchoscopy comparisons, t-tests were applied for continuous variables, and the Wilcoxon signed-rank test was used for ordinal data. Missing follow-up data were handled via the last observation carried forward (LOCF) method. Median OS, PFS, and 3-, 5-, and 10-year survival rates were estimated using the Kaplan-Meier method. Intergroup differences were assessed with the log-rank test, and survival curves were generated. All variables that had a p value < 0.50 on univariate analyses were considered as candidate variables for multivariate models. Multivariate analysis was conducted using the Cox proportional hazards model to calculate hazard ratios (HRs) with 95% confidence intervals (CIs). A two-sided p < 0.05 was considered statistically significant.

## 3 Results

### 3.1 Baseline clinical characteristics of TACC patients

A total of 52 TACC patients were included in this study, comprising 25 males and 27 females, with a mean age of 51.77 ± 14.40 years. 15 patients (28.85%) had a history of smoking. Dyspnea was the predominant initial symptom (40/52, 76.92%), followed by hemoptysis (7/52, 13.46%), cough (3/52, 5.77%), and incidental findings during physical examination (2/52, 3.85%). Additionally, 10 patients (19.23%) received surgical lesion resection, 25 patients (48.08%) underwent chemoradiotherapy, and only 25 patients (48.08%) were managed exclusively with interventional bronchoscopy during their disease course (**Table 1**).

**Table 1:** Demographic data and baseline characteristics of TACC patients.

Characteristics	Number of patients (%) (*n* = 52)
Gender	
Male	25 (48.08%)
Female	27 (51.92%)
Mean age (years)	51.77 ± 14.40
Body mass index (kg/m²)	23.32 ± 3.87
Smoking history	15 (28.85%)
Comorbidities	
Concurrent tumors	7 (13.46%)
Hypertension	13 (25%)
Initial symptom	
Dyspnea	40 (76.92%)
Hemoptysis	7 (13.46%)
Cough	3 (5.77%)
Incidental findings	2 (3.85%)
Lung involvement	18 (34.62%)
Treatment	
Bronchoscopic intervention	52 (100%)
Surgery	10 (19.23%)
Bronchoscopic intervention alone	25 (48.08%)
Chemoradiotherapy	25 (48.08%)

### 3.2 Features of bronchoscopic intervention in TACC patients

Notably, all patients developed varying degrees of dyspnea upon presentation to our center, with 66.67% (26/39) achieving an mMRC score of 4, necessitating multiple bronchoscopic interventions. The mean degree of stenosis was 77.18 ± 14.95%, and the mean length was 3.80 ± 2.21 cm. Intraluminal stenosis was observed in 59.62% (31/52) of patients, while 40.38% (21/52) exhibited mixed stenosis type, endoscopically. Freitag grade IV or higher was documented in 61.22% (30/49) of cases, including one patient with nearly complete airway obstruction requiring emergency bronchoscopic intervention. Lesions predominantly involved the central airways (48/52, 92.31%), with or without peripheral bronchial involvement. A total of 500 interventional bronchoscopy procedures were performed across all patients, with a mean of 9.62 ± 7.71 sessions per patient. To maximally alleviate symptoms and airway stenosis, bronchoscopists employed multimodal approaches tailored to individual clinical needs: stent implantation was performed in 42.31% (22/52) of patients, balloon dilation in 23.08% (12/52), thermal ablation (electrocautery, laser or APC) in 90.38% (47/52), and cryoablation in 73.08% (38/52) (**Table 2**).

**Table 2:** Features of bronchoscopic intervention in TACC patients.

Procedural characteristics	Results
Number of interventional procedures	500
Anesthesia methods (*n* = 500)	
General anesthesia	253 (50.60%)
Local anesthesia	247 (49.40%)
Respiratory support modalities (*n* = 500)	
Jet ventilation via rigid bronchoscopy	39 (7. 80%)
Endotracheal intubation	29 (5.80%)
laryngeal mask	166 (33.20%)
Mask	4 (0.80%)
Nasal cannula	265 (53.00%)
mMRC score (*n* = 52)	
2	2 (3.85%)
3	17 (32.69%)
4	33 (63.46%)
Stenosis location (*n* = 52)	
Upper trachea	12 (23.08%)
Mid trachea	19 (36.54%)
Lower trachea	17 (32.69%)
Left main bronchus	5 (9.62%)
Right main bronchus	5 (9.62%)
Stenosis length (cm) (*n* = 52)	3.80 ± 2.21
Stenosis type (*n* = 52)	
Intraluminal	31 (59.62%)
Mixed	21 (40.38%)
Stenosis degree% (CT) (*n* = 49)	77.18 ± 14.95
Freitag grade (*n* = 49)	
II	2 (4.08%)
III	17 (34.69%)
IV	21 (42.86%)
V	9 (18.37%)
Bronchoscopic interventional modalities (*n* = 52)	
Stent implantation	22 (42.31%)
Balloon dilation	12 (23.08%)
Thermal ablation (electrocautery, laser or APC)	47 (90.38%)
Cryoablation	38 (73.08%)

### 3.3 Short-term efficacy and safety of bronchoscopic intervention in TACC patients

**Figure 1:**
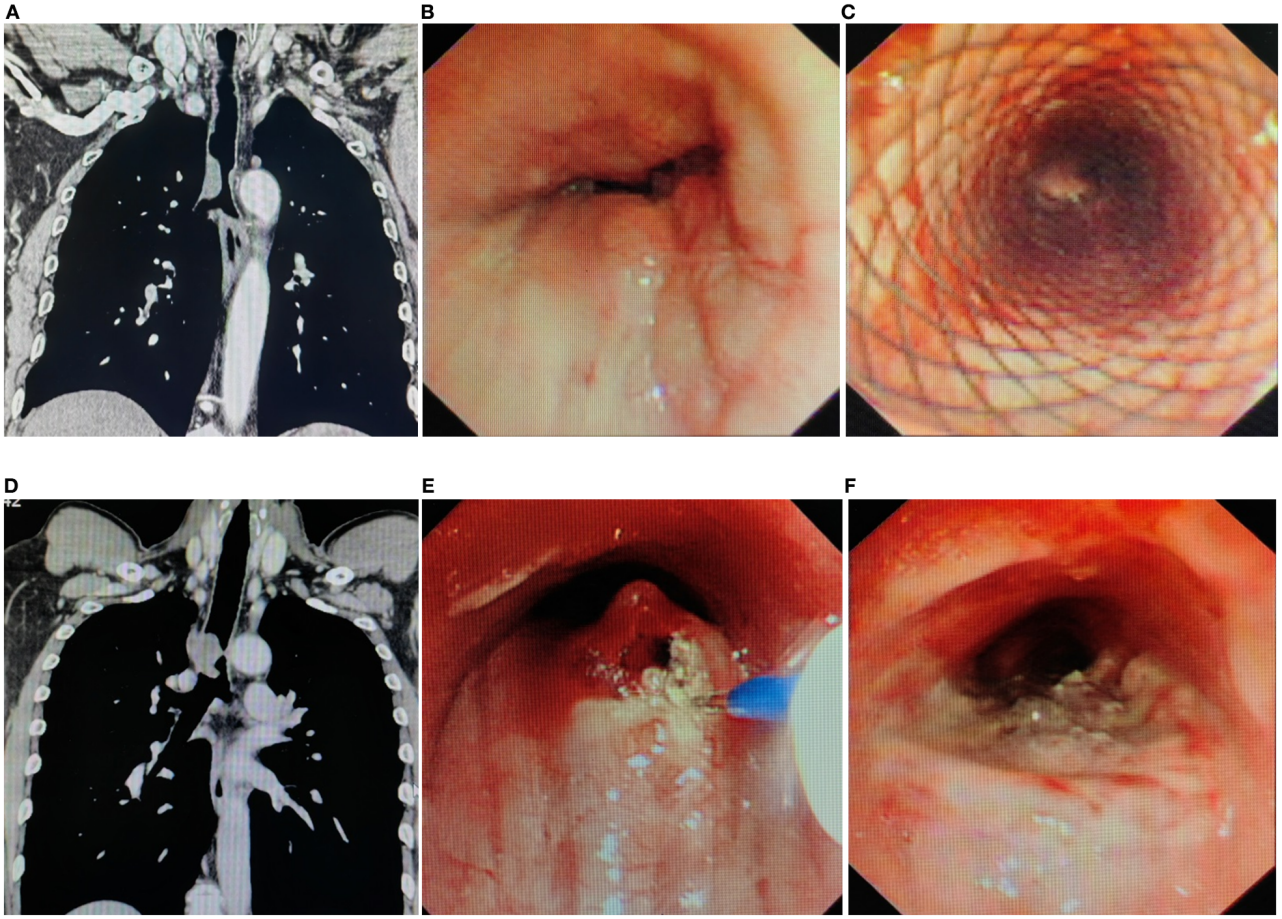
Imaging and endoscopic features of airway stenosis in TACC patients **(A, B)** Coronal chest CT and endoscopic images of a TACC patient (male/64ys) demonstrating Intraluminal type tracheal stenosis. **(C)** Post-interventional endoscopic view showing airway stent implantation with resolution of obstruction. **(D, E)** Coronal chest CT and endoscopic images of another TACC patient (male/56ys) with Intraluminal stenosis. **(F)** Post- interventional endoscopic view after electrosurgical snare resection and APC ablation, demonstrating significant improvement in stenosis.

**Figure 2:**
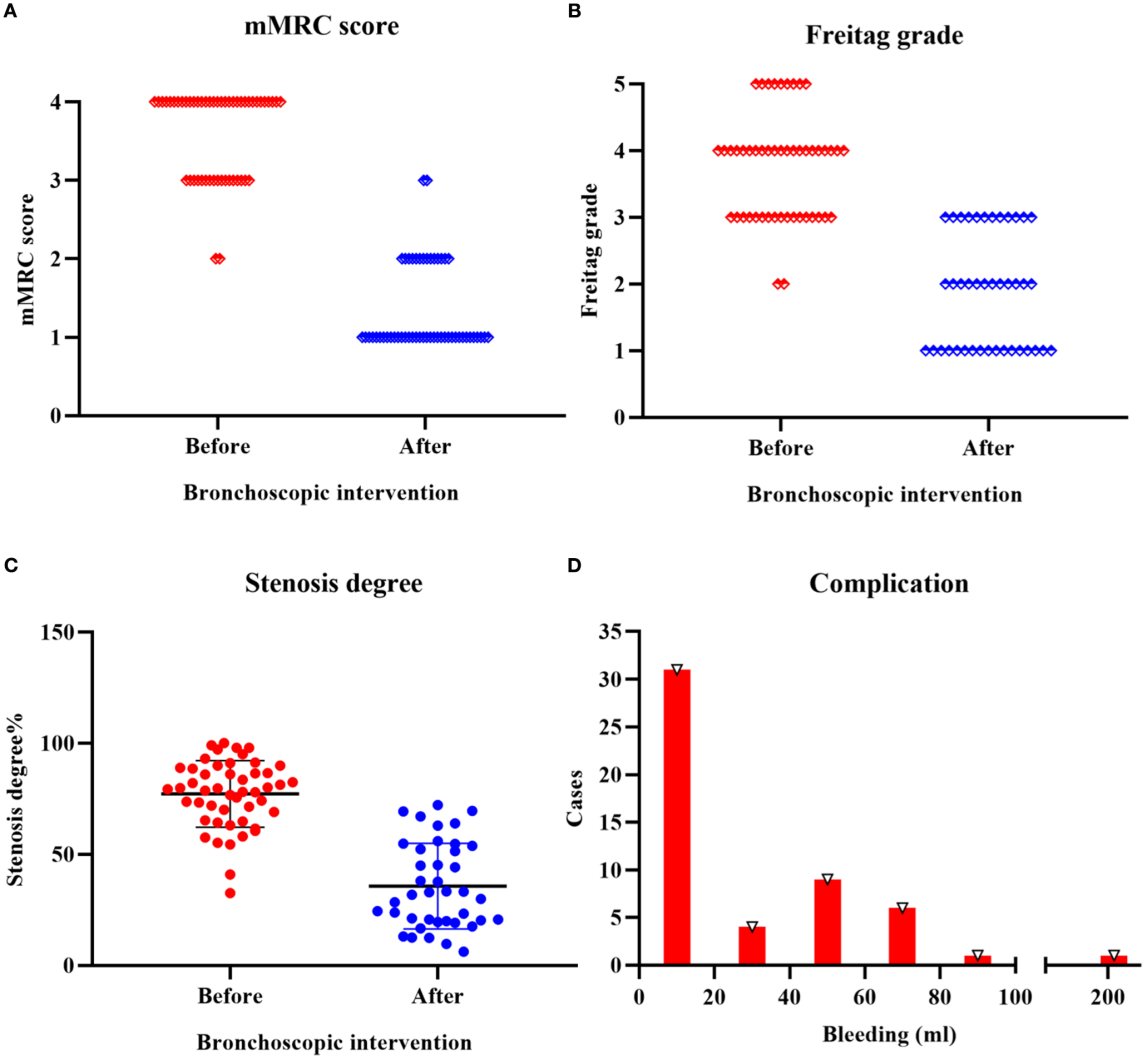
Efficacy and safety of bronchoscopic intervention in TACC patients **(A)** Significant reduction in mMRC score following bronchoscopic intervention (*P* < 0.01). **(B)** Post-interventional improvement in Freitag grade (*P* < 0.01). **(C)** Decrease in mean airway stenosis degree from 77.18 ± 14.95% pre-treatment to 35.64 ± 18.99% post-treatment (*P* < 0.01). **(D)** Intraoperative hemorrhage severity distribution: < 20 mL in 31 patients, 20–40 mL in 4 patients, 40–60 mL in 9 patients, 60–80 mL in 6 patients, and 80–100 mL in 1 patient. One patient experienced significant hemorrhage exceeding 200 mL (> 100 mL).

All TACC patients underwent interventional bronchoscopy for airway stenosis (**Figure 1**). Compared to pre-treatment values, post-interventional mMRC scores significantly decreased (*P* < 0.01) (**Figure 2A**, **Table 3**), as did Freitag grade (*P* < 0.01) (**Figure 2B**, **Table 3**). The mean airway stenosis degree improved from 77.18 ± 14.95% pre-treatment to 35.64 ± 18.99% post-treatment (*P* < 0.01) (**Figure 2C**, **Table 3**), demonstrating marked alleviation of dyspnea and stenosis. Hemorrhage was the predominant intraoperative complication. Intraoperative blood loss was categorized as follows: < 20 mL in 31 patients, 20–40 mL in 4 patients, 40–60 mL in 9 patients, 60–80 mL in 6 patients, and 80–100 mL in 1 patient. One patient experienced significant hemorrhage exceeding 200 mL (> 100 mL) (**Figure 2D**). All bleeding episodes were successfully managed with endoscopic hemostatic interventions (e.g., topical epinephrine, APC). No procedure-related mortality or fatal complications occurred.

**Table 3:** Short-term efficacy of bronchoscopic intervention in TACC patients.

Variables	Pre-treatment (number of patients)	Post-treatment (number of patients)	Statistics	*P* values
mMRC score	*n* = 52	*n* = 52	*Z* = -8.942	*P* < 0.001
1	0	36		
2	2	14		
3	17	2		
4	33	0		
Freitag grade	*n* = 49	*n* = 41	*Z* = -7.231	*P* < 0.001
I	0	17		
II	2	12		
III	17	12		
IV	21	0		
V	9	0		
Stenosis degree%	77.18 ± 14.95 (*n* = 49)	35.64 ± 18.99 (*n* = 41)	*T* = 14.07	*P* < 0.001

### 3.4 Role of bronchoscopic intervention in long-term management of TACC patients

**Figure 3:**
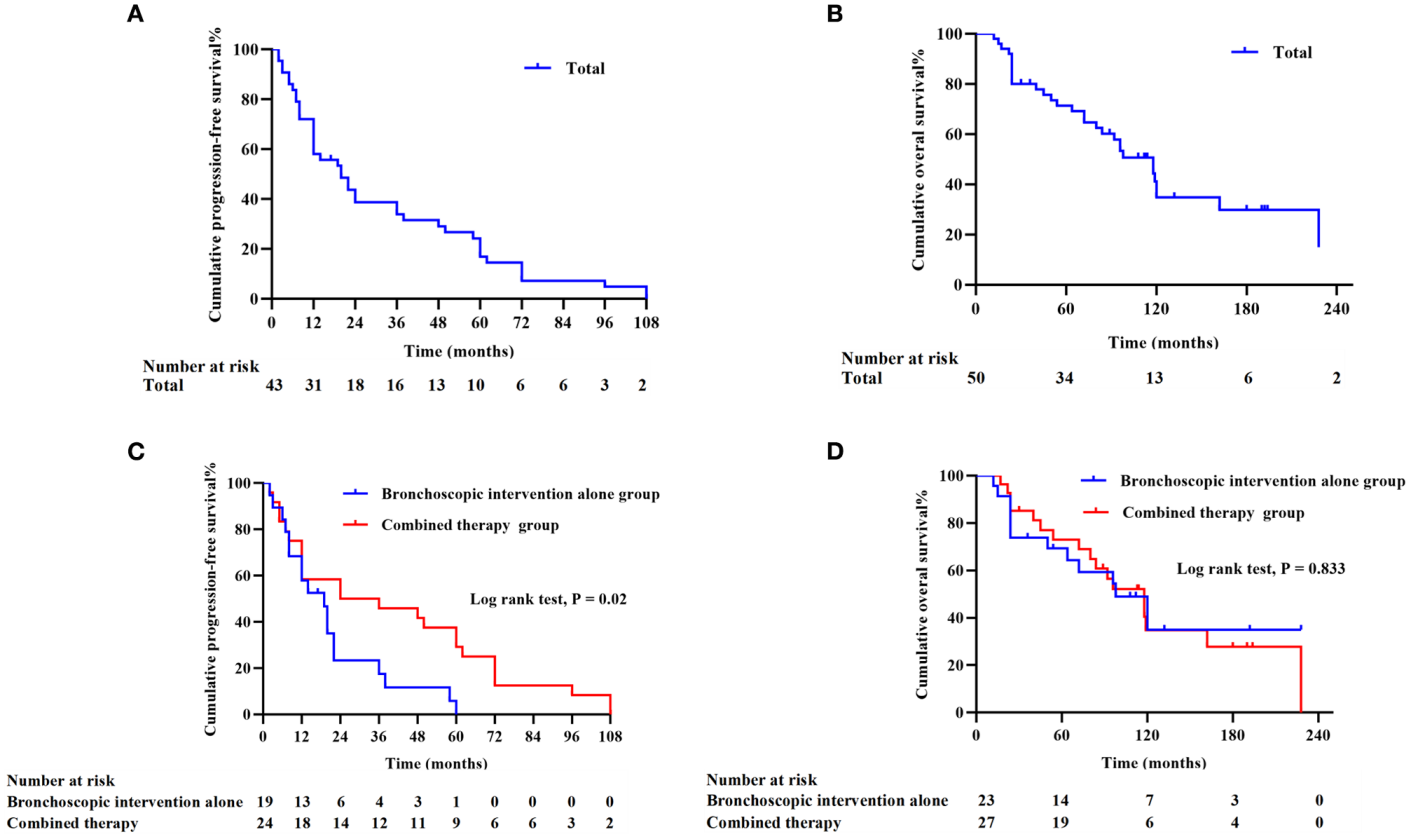
Survival outcomes in TACC patients (missing clinical data in individual cases) **(A)** Progression-free survival (PFS) curve for 43 TACC patients. **(B)** Overall survival (OS) curve for 50 TACC patients. **(C)** Comparison of PFS between the bronchoscopic intervention alone group (*n* = 19) and combined therapy group (*n* = 24). The combined therapy group demonstrated significantly superior PFS (*P* < 0.05). **(D)** OS comparison between the bronchoscopic intervention alone group (*n* = 23) and combined therapy group (*n* = 27), showing no statistically significant difference (*P* = 0.833).

To evaluate the long-term survival and prognostic value of interventional bronchoscopy in TACC patients, Kaplan-Meier analysis was performed to estimate median survival time and survival rates. After excluding cases with incomplete clinical data, the median PFS was 20 months in 43 patients, and the median OS was 118 months in 50 patients. The 3-year and 5-year PFS rates were 33.97% and 17.00%, respectively (**Figure 3A**), with the longest PFS reaching 108 months. The 3-year, 5-year, and 10-year OS rates were 80.00%, 69.12%, and 34.87%, respectively (**Figure 3B**). To assess the impact of treatment modalities on outcomes, patients were stratified into two groups: the bronchoscopic intervention alone group (*n* = 25, managed exclusively with interventional bronchoscopy) and combined therapy group (*n* = 27, receiving bronchoscopy plus surgical resection and/or chemoradiotherapy). The combined therapy group demonstrated significantly prolonged median PFS (30 months *vs*. 19 months; *HR* = 0.633, 95% CI: 0.344 – 1.167, *P* = 0.02) (**Figure 3C**). However, no statistically significant difference in median OS was observed between the combined therapy group (118 months) and the bronchoscopic intervention alone group (98 months) (*HR* = 0.831, 95% CI: 0.403 – 1.710, *P* = 0.833) (**Figure 3D**).

**Figure 4:**
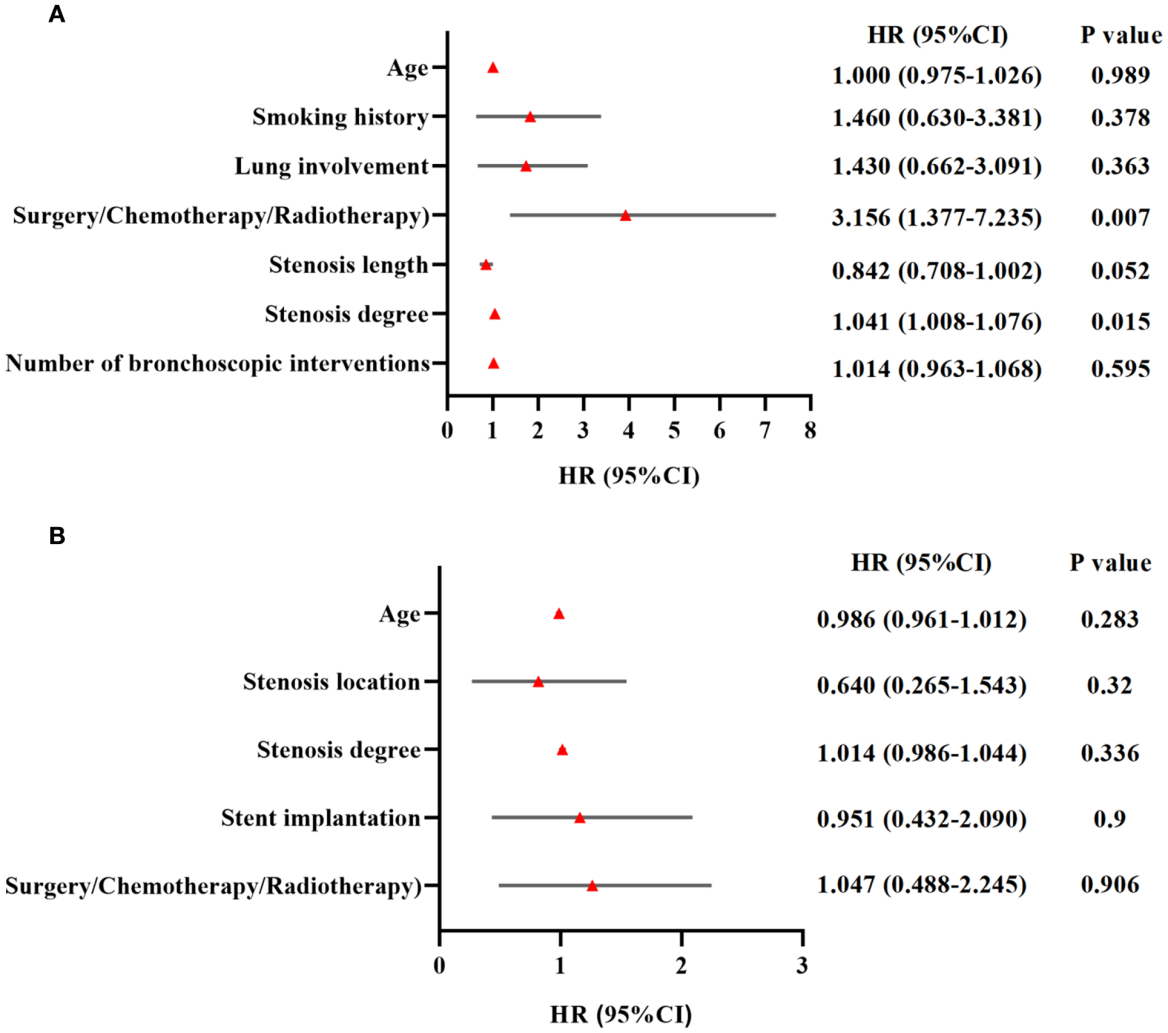
Multivariate cox proportional hazards regression analysis of clinical factors influencing prognosis in TACC patients **(A)** Combined therapy (Bronchoscopy/surgery/chemotherapy/radiotherapy) and stenosis degree were identified as independent clinical factors influencing progression-free survival (PFS) (*P* < 0.05). **(B)** No clinical factors demonstrated statistically significant associations with overall survival (OS) (*P* > 0.05).

Univariate Cox regression analysis was conducted to identify clinical factors influencing PFS and OS, including sex, age, BMI, smoking history, lung involvement, combined therapy, stenosis location, stenosis length, stenosis type, stenosis degree, stent implantation and number of interventional procedures. Then, candidate variables in univariate analysis were included in multivariate Cox regression. Multivariate analysis revealed that combined therapy (*HR* = 3.156, 95% CI: 1.377 – 7.235, *P* = 0.007) and stenosis degree (*HR* = 1.041, 95% CI: 1.008 – 1.076, *P* = 0.015) were independent predictors of PFS (**Figure 4A**). No factors showed statistically significant associations with OS (*P* > 0.05) (**Figure 4B**).

## 4 Discussion

TACC is a rare malignancy, recognized as the second most common subtype among bronchial tumors (17, 18). It is characterized by insidious growth, locally aggressive behavior, and a propensity for recurrence (2). This study presents the first systematic review evaluating both the short-term therapeutic efficacy and long-term prognostic implications of interventional bronchoscopy in TACC management, establishing its critical role within palliative multidisciplinary care frameworks. In this study, analysis of bronchoscopic intervention in 52 TACC patients demonstrated its critical role in alleviating airway obstruction. Long-term follow-up revealed that combining interventional bronchoscopy with surgical resection or chemoradiotherapy enhanced patient prognosis, further underscoring the importance of multidisciplinary management in TACC. However, the tumor’s invasive nature necessitated repeated bronchoscopic interventions to sustain long-term patency, highlighting the pivotal value of interventional bronchoscopy in palliative care.

In this study, the 52 TACC patients had a mean age of 51.77 years with a nearly equal male-to-female ratio (1:1). Dyspnea was the most common presenting symptom, followed by cough, hemoptysis, and incidental detection during routine physical examination in a minority of cases. A 2021 systematic review by Juntao Ran et al. (19), which analyzed 76 articles involving 1,252 TACC patients, reported a mean age of 48.70 years, a male-to-female ratio of 1:1.17, and dyspnea in 86% of cases. Our findings align with these reports, indicating that TACC predominantly affects middle-aged individuals without significant gender predilection and that dyspnea is the hallmark clinical manifestation due to the tumor’s predilection for the central airways. Notably, only 28.85% of our cohort had a smoking history, a proportion markedly lower than the strong smoking association observed in typical airway squamous cell carcinoma. This suggests that ACC pathogenesis is less correlated with tobacco exposure (17) and may instead be driven by molecular genetic aberrations, such as the MYB-FIB fusion gene, as previously reported (20).

In this study, 63.46% patients presented with an mMRC score of 4 upon presentation to our center, and the mean airway stenosis degree approached 80%, with 61.22% classified as Freitag grade IV or higher. These findings indicate that the majority of enrolled patients had severe central airway obstruction, necessitating urgent bronchoscopic intervention to preserve respiratory function (8, 21). Such stenosis is closely associated with TACC’s characteristic circumferential infiltration along the tracheobronchial wall. Regarding anesthesia and ventilation management, general anesthesia was utilized in 50.60% of procedures, with artificial airway establishment recommended through laryngeal mask, rigid bronchoscopy, or endotracheal intubation, likely reflecting the need for stable ventilation during complex interventions such as stent implantation or extensive ablation. Therapeutic approaches included thermal ablation (electrocautery, laser, or APC) in 90.38% of patients, often combined with cryotherapy (73.08%). Thermal ablation and cryorecanalization enables rapid debulking of intraluminal tumor tissue (22–24), while cryotherapy inhibits deep invasion by disrupting tumor microvasculature and inducing apoptosis (25, 26). Their synergistic use may delay disease recurrence. Airway stents were implanted in 42.31% of patients, and balloon dilation was performed in 23.08%. Levent Dala et al. reported stent implantation in 147 of 547 patients with malignant airway stenosis (27), underscoring the need for mechanical support in cases of cartilage destruction or extrinsic compression (21). However, vigilance is required for stent-related complications such as granulation hyperplasia or migration (21, 28). Thus, a multimodal bronchoscopic strategy is essential to maintain long-term airway patency in malignant stenosis (29).

This study confirmed that interventional bronchoscopy significantly alleviates airway obstruction in TACC patients, with marked reductions in post-interventional mMRC scores, Freitag grade, and stenosis degree compared to baseline. These findings underscore the efficacy of bronchoscopic interventional techniques in rapidly resolving critical airway compromise, consistent with prior studies (8, 10, 30). While surgical resection is preferred for operable ACC, interventional bronchoscopy provides crucial symptomatic and oncologic control for unresectable disease within an MDT framework (e.g., thoracic surgery, oncology, etc). Prospective trials should define its neoadjuvant/adjuvant roles. Intraoperative hemorrhage was the predominant procedural risk, with all bleeding events successfully managed via endoscopic hemostasis and no procedure-related mortality, demonstrating a well-controlled safety profile. Notably, one patient experienced substantial hemorrhage (200mL) during her procedure involving electrocautery (snare and probe) and cryotherapy. This excessive bleeding may correlate with high tumor burden, hypervascularity, and mechanical techniques, including large biopsy forceps and cryoresection. Thus, high-risk interventions warrant pre-hemostatic measures, such as prophylactic endobronchial balloon placement or APC ablation of the tumor base. In this cohort, 40.38% of patients had intraoperative blood loss exceeding 20 mL, a rate notably higher than previously reported in bronchoscopic interventions for lung cancer (13, 31–33). This discrepancy may stem from ACC’s aggressive invasion, which disrupts submucosal microvasculature (2). Nevertheless, advancements in operator experience and novel hemostatic techniques (e.g., APC, prophylactic endobronchial balloon) hold promise for mitigating severe hemorrhage risks (34).

Our survival analysis further elucidates the role of interventional bronchoscopy in the comprehensive management of TACC. Despite its indolent growth, TACC frequently recurs, necessitating repeated bronchoscopic intervention to alleviate dyspnea and airway stenosis for survival maintenance. In our cohort, a total of 500 interventional bronchoscopy procedures were performed across all patients, with a mean of 9.62 sessions per patient. The median PFS was 20 months, and median OS reached 118 months, with 5- and 10-year survival rates of 69.12%, and 34.87%, respectively. Hongwu Wang et al. (8) reported comparable 5- and 10-year survival rates (85.9% and 45.9%) in 37 TACC patients managed with repeated interventional bronchoscopy, demonstrating outcomes non-inferior to surgical resection. This underscores the pivotal role of bronchoscopy in long-term TACC management. Subgroup analysis revealed significantly prolonged median PFS in the combined therapy group compared to the bronchoscopic intervention alone group, likely attributable to ACC’s radio sensitivity and tumor burden reduction via surgical resection (4, 35, 36). Multivariate Cox regression identified combined therapy and mild-to-moderate stenosis degree as independent protective factors for PFS, suggesting early integration of systemic therapies (e.g., radiotherapy) may delay local recurrence. Similarly, Yalong Wang et al. (37) observed superior 5- and 10-year survival rates in surgically resected TACC patients (85.00% and 63.40% vs. 63.70% and 46.40% in non-surgical cohorts), further supporting the survival advantage of multimodal approaches. Our findings of this study demonstrate a relatively low survival rate, potentially associated with the enrollment of patients presenting with more severe clinical conditions, which may indicate the presence of selection bias. Notably, while combined therapy significantly improved PFS, OS benefits did not reach statistical significance, implying that surgical/chemoradiotherapy efficacy diminishes over time with disease progression. Long-term survival may ultimately depend on bronchoscopic palliation to relieve airway obstruction and dyspnea (38). The absence of independent OS predictors in multivariate analysis may reflect limited sample size or follow-up duration.

It is evident that this study has several limitations. First, the small sample size and single-center retrospective design may restrict the statistical power and introduce selection bias, particularly given the predominance of patients with severe stenosis (Freitag grade IV or higher) requiring urgent intervention. Moreover, treatment selection between groups was also influenced by non-medical confounders (e.g., socioeconomic status, healthcare access, patient preference), which are difficult to quantify and adjust for retrospectively. These imbalances introduce bias that might affect outcome interpretation. Non-uniform treatments reflect real-world ACC management constraints. While this limits direct comparisons, it captures actual clinical decision-making. Future prospective studies should standardize protocols where feasible. Second, heterogeneity in bronchoscopic techniques and operator experience across institutions could overestimate the real-world efficacy of therapeutic interventions. Additionally, the use of the last observation carried forward (LOCF) method for missing data imputation may introduce potential bias. Future multicenter prospective studies incorporating molecular subtyping (e.g., MYB-FIB fusion status) and imaging assessments of stenosis progression are warranted to refine personalized therapeutic strategies. Furthermore, exploring synergistic effects between bronchoscopic interventions and systemic therapies—such as anti-angiogenic agents or immune checkpoint inhibitors—may offer novel avenues to improve long-term outcomes (39, 40).

In conclusion, for TACC patients with severe airway stenosis, interventional bronchoscopy is recommended as the cornerstone intervention to relieve airway obstruction. Early integration with surgical resection and/or chemoradiotherapy is critical to delay disease progression. Long-term prognostic management of TACC requires a balanced approach between local control and systemic therapy. By maintaining durable airway patency, interventional bronchoscopy provides a vital platform for multimodal treatment opportunities, ultimately enhancing patients’ survival.

## 5 Conclusions

Bronchoscopic intervention demonstrated significant efficacy in alleviating dyspnea and airway stenosis while sustaining long-term survival in TACC patients. Multimodal therapy provided incremental benefits in delaying disease progression.

## Data Availability

The original contributions presented in the study are included in the article/supplementary material. Further inquiries can be directed to the corresponding authors.
